# Fluorescent labeling of NASBA amplified tmRNA molecules for microarray applications

**DOI:** 10.1186/1472-6750-9-45

**Published:** 2009-05-15

**Authors:** Ott Scheler, Barry Glynn, Sven Parkel, Priit Palta, Kadri Toome, Lauris Kaplinski, Maido Remm, Majella Maher, Ants Kurg

**Affiliations:** 1 Dept. of Biotechnology, Institute of Molecular and Cell Biology, University of Tartu, Tartu, Estonia; 2 Estonian Biocentre, Tartu, Estonia; 3 Molecular Diagnostics Research Group, National Centre for Biomedical Engineering Science, National University of Ireland, Galway, Ireland

## Abstract

**Background:**

Here we present a novel promising microbial diagnostic method that combines the sensitivity of Nucleic Acid Sequence Based Amplification (NASBA) with the high information content of microarray technology for the detection of bacterial tmRNA molecules. The NASBA protocol was modified to include aminoallyl-UTP (aaUTP) molecules that were incorporated into nascent RNA during the NASBA reaction. Post-amplification labeling with fluorescent dye was carried out subsequently and tmRNA hybridization signal intensities were measured using microarray technology. Significant optimization of the labeled NASBA protocol was required to maintain the required sensitivity of the reactions.

**Results:**

Two different aaUTP salts were evaluated and optimum final concentrations were identified for both. The final 2 mM concentration of aaUTP Li-salt in NASBA reaction resulted in highest microarray signals overall, being twice as high as the strongest signals with 1 mM aaUTP Na-salt.

**Conclusion:**

We have successfully demonstrated efficient combination of NASBA amplification technology with microarray based hybridization detection. The method is applicative for many different areas of microbial diagnostics including environmental monitoring, bio threat detection, industrial process monitoring and clinical microbiology.

## Background

There is a growing need for quick and highly parallel approaches to microorganism detection and identification worldwide. In this paper we present a molecular diagnostics method that combines isothermal amplification of target tmRNA molecule with microarray-based detection. Nucleic Acid Sequence Based Amplification (NASBA) is a sensitive isothermal RNA amplification technology [1] that offers several advantages over the more commonly used PCR-based methods: it requires simplified equipment, is less sensitive to genomic DNA contamination and therefore more suitable for applications where microbial viability is important [2]. In microbial diagnostics, NASBA has been successfully combined with electrochemiluminescent (ECL) [3] ELISA [4] labeled dendrimer [5] and molecular beacon-based [6] methods to detect and identify viral and bacterial pathogens. DNA microarrays have recently shown great potential in microbial diagnostics; in investigation of microbial diversity, composition and species identification from environmental and medical samples [7,8]. In microarray experiments amplified nucleic acid sequences of interest are usually labeled prior to the hybridization experiment. Use of fluorescent labeling protocols for different amplification methods have previously been described [9-12], but to our knowledge no method has been published for direct labeling of NASBA amplified products. Previously published NASBA product labeling methods like NASBA-ELISA [4] and NAIMA [5] all require additional secondary enzymatic steps to complete the protocol. Introduction of microarray based detection to NASBA offer the new potential for rapid simultaneous identification of many different amplification products and/or pathogens. Delicate and precise reaction conditions have to be present for NASBA amplification of target RNA with high efficiency, as the method requires simultaneous cooperation and balance between three different enzymes (reverse transcriptase, RNaseH and T7 RNA polymerase) and their respective buffers. Any further modifications to the gold standard NASBA protocol (like the one developed by bioMerieux, patent holder for NASBA technology) for microarray purposes, require cautious approach and carefully conducted experiments in order to maintain adequate RNA amplification yield. The objective of our work is to present a method that combines simple one-step fluorescent labeling of NASBA amplified tmRNA product with microarray hybridization and detection. TmRNA products of bacterial *ssrA *genes were used as markers because they contain regions of sequence heterogenecity that have previously been successfully applied for microbial diagnostics [13,14]. Aminoallyl modified UTPs (aaUTPs) were incorporated into nascent RNAs in the NASBA reaction. Following the amplification process, aaUTP modified RNAs were labeled with aminoreactive fluorescent marker Cy3. Fluorescently labeled tmRNA was then used in microarray hybridization experiments. Optimal concentrations for two different aaUTP-salts in NASBA reaction were determined.

## Results and Discussion

**Figure 1:**
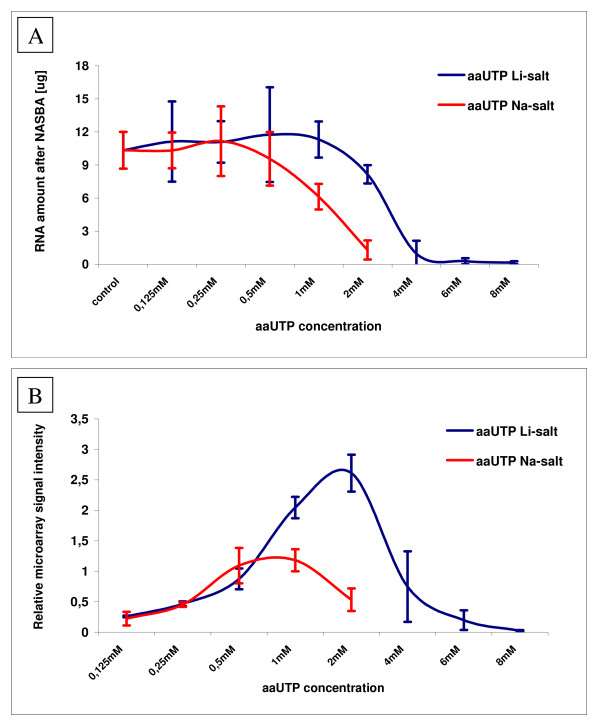
**Results of *Streptococcus pneumoniae *tmRNA specific NASBA amplifications**. A) Average RNA quantity after NASBA amplification with different aaUTP salts. Error bars show ± one SD of these averages. Control value stands for RNA amount in NASBA reaction without aaUTP addition. B) Comparison of average relative microarray signal intensities from NASBA experiments conducted with a range of concentrations of two different aaUTP. Error bars show ± one SD of these averages. Signal intensity value 1 stands for average signal intensity of every dilution series experiment including data from both used aaUTP salts.

Addition of aaUTP to NASBA mix resulted in a concentration-dependent effect on the reaction performance. Concentrations of up to 0,5 mM for sodium salt and 1 mM for lithium salt did not influence NASBA efficiency as seen from the amount of RNA produced (Fig. 1A), whereas higher concentrations did inhibit the amplification efficiency. The exact ratio of aaUTP to rUTP (and to all the other nucleotides correspondingly) and its influence on NASBA reactions cannot be determined precisely as the manufacturer's protocol do not provide information about the composition of Reagent sphere (component of bioMerieux NASBA kit containing NTP and dNTP molecules). Fig. 1A shows the average amount of RNA product generated with amplification reactions comparing aaUTP sodium salt to the aaUTP lithium salt. Specific amplification of *S*. pneumoniae tmRNA molecules was verified by observing only one peak of predicted size (307 nucleotides) nucleic acid on RNA 6000 chip electropherogram. Respective microarray signal intensities of labeled NASBA products are shown on Fig. 1B. Data for aautp lithium salt concentrations from 0,125 mM to 8 mM and for aaUTP sodium salt concentrations from 0,125 mM to 2 mM are given, respectively. Increased microarray signal intensity was observed in parallel with increasing aminoallyl-UTP concentration in NASBA reaction up to 1 mM for sodium and 2 mM for lithium salt. For aaUTP lithium salt the final concentrations within the range of 1 mM and 2 mM resulted in the highest average microarray signals, while highest average signals with aaUTP sodium salt were obtained between 0,5 mM and 1 mM aaUTP concentration, respectively. The final 2 mM concentration of aaUTP Li-salt in NASBA reaction resulted in highest microarray signals overall, being twice as high as the strongest signals with 1 mM aaUTP Na-salt.

The aim of this work was to improve and expand the potential of a combined NASBA-microarray approach by developing a new optimized protocol suitable for further possible use in microbial diagnostics. All together, we have introduced a new method suitable for high-speed parallel detection of RNA targets. NASBA technology, widely used in various microbiology laboratories all over the world, is for the first time combined with microarray based RNA detection technology using simple one-step NASBA product labeling method. Previous methods described so far for NASBA amplicon detection have used additional enzymatic steps after the amplification and secondary labeled probes [4,5]. In our case, the NASBA protocol was modified by addition of aminoallyl-UTP molecules allowing labeling of the reaction product with aminoreactive fluorescent marker. Indirect labeling of RNA for microarray purposes via incorporation of aaUTPs was preferred over direct incorporation of fluorescently labeled nucleotides, as it has been proved to be more efficient with T7 RNA polymerase based methods [12]. Two different aaUTP salts were compared and both enabled sufficient fluorophore incorporation providing easily detectable microarray signals. Considering that aminoallyl-UTPs tested in the current report are produced as different salts, this may contribute to the difference in RNA quantity in NASBA reaction (Fig. 1A) causing decrease in microarray signal intensities (Fig. 1B). The aaUTP Na-salt may have more negative impact than the Li-salt aaUTP on the co-operation of the NASBA enzymes. Different monovalent cations have previously been shown to have unequal impact on similar enzymatic reactions [15]. However, as the manufactures do not provide exact composition of aaUTP storage solution, other unknown components may also contribute to the observed difference in behaviour of used aminoallyl-UTPs.

## Conclusion

In general we have presented here a new optimized protocol for highly potential microarray based detection of NASBA amplified RNA products. Our method is suitable for further possible diagnostic applications where rapid identification of various RNA molecules from bacteria or different microorganisms is needed. The combination of NASBA with microarray detection is particularly advantageous in settings where different bacterial species may be present (such as environmental samples) or in clinical settings where it is necessary to identify one particular infection causing species from a panel of potential pathogens.

## Methods

### Bacterial strain and total RNA purification

*Streptococcus pneumoniae *strain ATCC 33400 (DSMZ, Braunschweig, Germany) was grown in Brain Heart Infusion Broth (Oxoid, Hampshire, UK). Total RNA was purified from overnight cultures using RiboPure Yeast Kit (Ambion, Austin, TX, USA). Although the RiboPure Yeast kit is not originally intended for RNA extraction from bacteria, high quality bacterial total RNA was obtained without making any modifications to manufacturer's instructions.

### NASBA

NASBA primer pair (Table 1) was designed to amplify near full-length 307 nucleotide tmRNA product from *S. pneumoniae *total RNA. Conventional NASBA, in which the T7 promoter is included in the reverse primer, generates an antisense RNA product [1]. In order to generate sense strand of tmRNA the T7 promoter sequence was positioned on the forward primer instead of the reverse, as described previously [16]. NASBA reactions were performed with 10 ng of template total RNA using NucliSENS EasyQ Basic Kit v2 (bioMerieux bv, Boxtel, NL) according to the manufacturer's instructions. Aminoallyl-UTP was added to the reaction mix (final volume 20 μl) at concentrations ranging from 0,125 mM to 8 mM. Equal volume of NASBA water (bioMerieux) was added to control experiment without any aaUTP, to set a baseline for determination of amplification efficiency. Two different aminoallyl-UTP solutions: aaUTP Li-salt (Epicentre, Madison, WI, USA) and aaUTP Na-salt (Fermentas, Vilnius, Lithuania), were tested separately to determine the optimal concentration range for both. Experiments were replicated at least three times.

**Table 1:** *Streptococcus pneumoniae *NASBA primers used in current experiment

Primer	Sequence 5'-> 3'
Forward +T7	AATTCTAATACGACTCACTATAGGGAGAAGGTTCGACAGGCATTATGAGGCATA
Reverse	CGTCCAAACACCTGCCAACATA

### NASBA product purification and labelling

Following amplification, NASBA products were purified with NucleoSpin RNA Clean-Up kit (Macherey-Nagel, Düren, Germany) and vacuum dried. Labeling of the RNA product was carried out using protocol published by 't Hoen and colleagues with some modifications [12]. The RNA pellet was resuspended in 8,5 μl 0,1 M Na_2_CO_3 _and incubated for one hour with 3 nmoles of amino reactive Cy3-*N*-hydroxysuccinimide ester dye (Enzo, Farmingdale, NY, USA) dissolved in 0,5 μl DMSO (Applichem, Darmstadt, Germany). Excess dye was quenched by addition of 3,5 μl 4 M hydroxylamine solution (Sigma-Aldrich, Steinheim, Germany). Labeled RNA was purified with NucleoSpin kit and quantified using Bioanalyzer 2100 platform with RNA 6000 Nano Chip (both Agilent, Santa Clara, CA, USA) according to the manufacturers manual.

### *S. pneumoniae *specific microarray

The custom made microarray contained 97 probes, covering whole *S. pneumoniae *tmRNA sequence (see Additional file 1). Probes were designed with the SLICSel 1.0 software . The average length of probes was 16 nucleotides, their average Tm was 58°C and average ΔG-23 kcal/mol (calculated by DNA-RNA thermodynamics). Probes were ordered from Metabion (Mariensried, Germany) and spotted onto SAL-1 Ultra microarray slides by Asper Biotech (Tartu, Estonia).

### Microarray hybridization

One third of the labeled NASBA product was resuspended in 80 μl of microarray hybridization buffer and hybridized for four hours on the *S. pneumoniae *specific microarray in an automated HS-400 hybridization station (Tecan Austria, Grödig, Austria) at 34°C. Complete hybridization protocol is shown in Table 2.

**Table 2:** Microarray hybridization protocol used in an automated HS-400 hybridization station

		**Temp. C**	**Duration**	**Repetitions**
**1**	Prewash	85	Wash: 60 s; Soak: 30 s	1

**2**	Probe injection	34		1

**3**	Hybridization	34	4 h 00 min, High agitation	1

**4**	1. wash	23	Wash: 90 s; Soak: 30 s	3

**5**	2. wash	23	Wash: 90 s; Soak: 30 s	3

**6**	3. wash	23	Wash: 90 s; Soak: 30 s	3

**7**	Slide drying	23	90 s	1

1. Prewash – 6× saline sodium citrate (SSC); 0,5% Na-dodecyl sulphate (SDS)

2. Hybridization – 6× SSC; 0,5% SDS and 5× Denhardts solution

3. 1. wash – 2× SSC; 0,03% SDS

4. 2. wash – 1× SSC

5. 3. wash – 0,2× SSC

### Microarray scanning and data analysis

Slides were scanned using Affymetrix 428 scanner (Santa Clara, CA, USA) λ = 532 nm. Raw signal intensity data was analyzed with Genorama software (Asper Biotech). Fixed concentration of short fluorescent oligonucleotide control sequences ('spikes'), complementary to the control oligonucleotides on microarray, were added to the final 80 μl hybridization solution for normalization purposes. At first step all signals from all microarrays, obtained from dilution series experiment, were rescaled by equating the average of spike-specific signals. Secondly, to compare data from 97 different probes on single microarray, all individual signal intensities were divided with the corresponding probe specific average signal intensity calculated over all microarrays in one replicate experiment. Average over probe-wise normalized signal intensities (relative microarray signal intensities) were then calculated for each dilution series point (for each single microarray). Finally, considering replicate experiments (at least three), confidence intervals (± one SD) were calculated for each average dilution series point (for every different aaUTP concentration).

## Authors' contributions

OS carried out the NASBA and microarray experiments, conducted statistical analysis and drafted the manuscript. BG carried out the microbiological experiments and RNA extraction and helped to draft the manuscript. SP participated in NASBA and microarray experiments and helped to draft the manuscript. KT participated in NASBA and microarray experiments. PP designed the microarray and helped to draft and review the manuscript. LK helped designing microarray and helped with data analysis. MR participated in design and coordination of making a microarray and helped conduct data analysis. MM conceived of the study and participated in its design and coordinated microbiological experiments. AK conceived of the study, participated in its design, coordinated NASBA and microarray experiments and helped to draft the manuscript. All authors read and approved the final manuscript.

## Supplementary Material

Additional file 1 **Microarray probes used in experiment**. Table showing all the 97 microarray probes (covering whole *S. pneumoniae *tmRNA sequence) and their characteristics. Click here for file
