# Supplementary material for: Fluorescent labeling of NASBA amplified tmRNA molecules for microarray applications
Source: BMC Biotechnol. 2009 May 15;9:45. doi: 10.1186/1472-6750-9-45 (PMC2685129; doi:10.1186/1472-6750-9-45)
Supplement: Additional file 1 — Microarray probes used in experiment. Table showing all the 97 microarray probes (covering whole S. pneumoniae tmRNA sequence) and their characteristics. [file 1472-6750-9-45-S1.doc]

| **Probe ID** | **Sequence** | **ΔG** | **Length** | **GC%** | **Tm** | **Position on tmRNA** |
| --- | --- | --- | --- | --- | --- | --- |
| Spne_1 | ATAATGCCTGTCGAA | -20,3 | 15 | 40 | 54,3 | 14-28 |
| Spne_2 | CATAATGCCTGTCGAA | -21,8 | 16 | 43,8 | 57,1 | 14-29 |
| Spne_3 | TCATAATGCCTGTCGA | -22,6 | 16 | 43,8 | 59,7 | 15-30 |
| Spne_4 | GTCGCAAAATATGCCT | -21,9 | 16 | 43,8 | 57,5 | 30-45 |
| Spne_5 | AGTCGCAAAATATGCCT | -23,3 | 17 | 41,2 | 59,3 | 30-46 |
| Spne_6 | GAGTCGCAAAATATGCC | -23,5 | 17 | 47,1 | 58,9 | 31-47 |
| Spne_7 | ACGAGTCGCAAAATATGC | -24,9 | 18 | 44,4 | 58,7 | 32-49 |
| Spne_8 | CGAGTCGCAAAATATGC | -23,1 | 17 | 47,1 | 55,9 | 32-48 |
| Spne_9 | ACACGAGTCGCAAAATATG | -25,5 | 19 | 42,1 | 58,8 | 33-51 |
| Spne_10 | CACGAGTCGCAAAATATG | -23,8 | 18 | 44,4 | 56,1 | 33-50 |
| Spne_11 | CACACGAGTCGCAAAATAT | -25,3 | 19 | 42,1 | 58,2 | 34-52 |
| Spne_12 | ACCTGCCAACATATT | -20,5 | 15 | 40 | 58,5 | 297-311 |
| Spne_13 | AACACCTGCC | -16,3 | 10 | 60 | 54,8 | 305-314 |
| Spne_14 | ACACGAGTCGCA | -19,1 | 12 | 58,3 | 53,4 | 40-51 |
| Spne_15 | TTTACGTCGCCACAC | -22,4 | 15 | 53,3 | 59,3 | 48-62 |
| Spne_16 | GTTTACGTCGCCACA | -21,9 | 15 | 53,3 | 57,2 | 49-63 |
| Spne_17 | CGTTTACGTCGCCACA | -23,9 | 16 | 56,2 | 59,3 | 49-64 |
| Spne_18 | GCGTTTACGTCGCCA | -23,2 | 15 | 60 | 59,2 | 51-65 |
| Spne_19 | AGCGTTTACGTCGCC | -23,1 | 15 | 60 | 58,6 | 52-66 |
| Spne_20 | GAGCGTTTACGTCGC | -22,3 | 15 | 60 | 55,9 | 53-67 |
| Spne_21 | TGAGCGTTTACGTCGC | -24,1 | 16 | 56,2 | 58,9 | 53-68 |
| Spne_22 | ACTGAGCGTTTACGTCG | -24,7 | 17 | 52,9 | 59,6 | 54-70 |
| Spne_23 | CTGAGCGTTTACGTCG | -22,9 | 16 | 56,2 | 56,6 | 54-69 |
| Spne_24 | AACTGAGCGTTTACGTC | -23,5 | 17 | 47,1 | 58,2 | 55-71 |
| Spne_25 | TTAACTGAGCGTTTACG | -22,2 | 17 | 41,2 | 55 | 57-73 |
| Spne_26 | TAGAGCGTAAGAAGTGTTAT | -24,9 | 20 | 35 | 55,6 | 89-108 |
| Spne_27 | AGAGCGTAAGAAGTGTTAT | -23,9 | 19 | 36,8 | 54,7 | 89-107 |
| Spne_28 | CTAGAGCGTAAGAAGTGTTAT | -26,3 | 21 | 38,1 | 58 | 89-109 |
| Spne_29 | GAGCGTAAGAAGTGTTA | -21,9 | 17 | 41,2 | 53,2 | 90-106 |
| Spne_30 | GCTAGAGCGTAAGAAGT | -23,8 | 17 | 47,1 | 58,9 | 94-110 |
| Spne_31 | AGCTAGAGCGTAAGAAG | -23,9 | 17 | 47,1 | 59,5 | 95-111 |
| Spne_32 | CAGCTAGAGCGTAAGA | -23,2 | 16 | 50 | 59,6 | 97-112 |
| Spne_33 | TGCTGGTTTTTAGGC | -21,2 | 15 | 46,7 | 57,2 | 112-126 |
| Spne_34 | CAAATCGGGTCACGC | -23,4 | 15 | 60 | 59 | 128-142 |
| Spne_35 | CCAAATCGGGTCACG | -23,2 | 15 | 60 | 58,8 | 129-143 |
| Spne_36 | AATCCAAATCGGGTCAC | -24,3 | 17 | 47,1 | 59,6 | 130-146 |
| Spne_37 | CAATCCAAATCGGGTCA | -24,1 | 17 | 47,1 | 59 | 131-147 |
| Spne_38 | GCAATCCAAATCGGGT | -23,4 | 16 | 50 | 59 | 133-148 |
| Spne_39 | AGCAATCCAAATCGGG | -23,6 | 16 | 50 | 59,6 | 134-149 |
| Spne_40 | GAGCAATCCAAATCGG | -22,6 | 16 | 50 | 57,9 | 135-150 |
| Spne_41 | CGAGCAATCCAAATCGG | -24,6 | 17 | 52,9 | 59,9 | 135-151 |
| Spne_42 | ACGCCTGCT | -15,8 | 9 | 66,7 | 53 | 123-131 |
| Spne_43 | CAGCTAGAGCGT | -18,2 | 12 | 58,3 | 52,1 | 101-112 |
| Spne_44 | ACGAGCAATCCAAATCG | -23,8 | 17 | 47,1 | 58,9 | 136-152 |
| Spne_45 | CACGAGCAATCCAAATC | -23,3 | 17 | 47,1 | 59,2 | 137-153 |
| Spne_46 | AACACGAGCAATCCAAAT | -24,2 | 18 | 38,9 | 59,9 | 138-155 |
| Spne_47 | ACACGAGCAATCCAA | -22 | 15 | 46,7 | 59,6 | 140-154 |
| Spne_48 | GAACACGAGCAATCC | -22 | 15 | 53,3 | 59,8 | 142-156 |
| Spne_49 | ATTGAACACGAGCAATC | -22,5 | 17 | 41,2 | 56,6 | 143-159 |
| Spne_50 | CATTGAACACGAGCAATC | -24 | 18 | 44,4 | 59 | 143-160 |
| Spne_51 | TCATTGAACACGAGCAAT | -24 | 18 | 38,9 | 59 | 144-161 |
| Spne_52 | GTCATTGAACACGAGC | -22,3 | 16 | 50 | 57,9 | 147-162 |
| Spne_53 | CTGTCATTGAACACGAG | -22,9 | 17 | 47,1 | 58,6 | 148-164 |
| Spne_54 | TGTCATTGAACACGAG | -21,5 | 16 | 43,8 | 55,4 | 148-163 |
| Spne_55 | CCTGTCATTGAACACG | -22,3 | 16 | 50 | 59,6 | 150-165 |
| Spne_56 | GACCTGTCATTGAAC | -20,3 | 15 | 46,7 | 57,4 | 153-167 |
| Spne_57 | AGACCTGTCATTGAAC | -21,8 | 16 | 43,8 | 59,5 | 153-168 |
| Spne_58 | AAGACCTGTCATTGA | -20 | 15 | 40 | 55,8 | 155-169 |
| Spne_59 | TCGCTAATAATAAGACC | -21,6 | 17 | 35,3 | 54,7 | 164-180 |
| Spne_60 | GTATCTCGCTAATAATAAGAC | -25,2 | 21 | 33,3 | 57,3 | 165-185 |
| Spne_61 | TATCTCGCTAATAATAAGAC | -23,9 | 20 | 30 | 56,2 | 165-184 |
| Spne_62 | ATCTCGCTAATAATAAGAC | -22,9 | 19 | 31,6 | 55,2 | 165-183 |
| Spne_63 | CGTATCTCGCTAATAATAAGAC | -27,2 | 22 | 36,4 | 58,9 | 165-186 |
| Spne_64 | CTCGCTAATAATAAGAC | -20,6 | 17 | 35,3 | 52,4 | 165-181 |
| Spne_65 | TTAATCGTATCTCGCTAATAATAAGA | -30,2 | 26 | 26,9 | 59,3 | 166-191 |
| Spne_66 | AATCGTATCTCGCTAATAATAAGA | -28,6 | 24 | 29,2 | 58,8 | 166-189 |
| Spne_67 | CTTAATCGTATCTCGCTAATAATAAG | -30,1 | 26 | 30,8 | 59,6 | 167-192 |
| Spne_68 | GCTTAATCGTATCTCGC | -23 | 17 | 47,1 | 57,6 | 177-193 |
| Spne_69 | GGCTTAATCGTATCTCG | -23 | 17 | 47,1 | 56,5 | 178-194 |
| Spne_70 | AAGGCTTAATCGTATCTCG | -25,3 | 19 | 42,1 | 58,7 | 178-196 |
| Spne_71 | CAAGGCTTAATCGTATCTC | -24,8 | 19 | 42,1 | 59 | 179-197 |
| Spne_72 | ACAAGGCTTAATCGTATCT | -24,8 | 19 | 36,8 | 59,1 | 180-198 |
| Spne_73 | GACAAGGCTTAATCGTATC | -24,9 | 19 | 42,1 | 58,7 | 181-199 |
| Spne_74 | AGACAAGGCTTAATCGTAT | -24,6 | 19 | 36,8 | 57,6 | 182-200 |
| Spne_75 | TTATCAAGCCGCTAG | -21,1 | 15 | 46,7 | 57 | 199-213 |
| Spne_76 | CTTATCAAGCCGCTA | -21,1 | 15 | 46,7 | 58,4 | 200-214 |
| Spne_77 | GCGAGTCTATCAATCT | -21,3 | 16 | 43,8 | 55,9 | 215-230 |
| Spne_78 | TGCGAGTCTATCAATCT | -23,1 | 17 | 41,2 | 59,1 | 215-231 |
| Spne_79 | CGAGTCTATCAATCT | -23,4 | 15 | 40 | 49,5 | 215-229 |
| Spne_80 | AACTGCGAGTCTATCAAT | -23,9 | 18 | 38,9 | 59,8 | 217-234 |
| Spne_81 | ACTGCGAGTCTATCA | -21,7 | 15 | 46,7 | 59,4 | 219-233 |
| Spne_82 | AAACTGCGAGTCTATC | -21,9 | 16 | 43,8 | 58 | 220-235 |
| Spne_83 | GAAACTGCGAGTCTAT | -21,7 | 16 | 43,8 | 57,6 | 221-236 |
| Spne_84 | AGAAACTGCGAGTCTAT | -23,2 | 17 | 41,2 | 59,4 | 221-237 |
| Spne_85 | TAGAAACTGCGAGTC | -21,2 | 15 | 46,7 | 56,9 | 224-238 |
| Spne_86 | CTAGAAACTGCGAGT | -20,9 | 15 | 46,7 | 56,8 | 225-239 |
| Spne_87 | TCTAGAAACTGCGAG | -21,4 | 15 | 46,7 | 59 | 226-240 |
| Spne_88 | GTCTAGAAACTGCGA | -21,2 | 15 | 46,7 | 58,3 | 227-241 |
| Spne_89 | AGTCTAGAAACTGCG | -21,1 | 15 | 46,7 | 57,3 | 228-242 |
| Spne_90 | TCGACACATAACTCAA | -21,5 | 16 | 37,5 | 56,9 | 242-257 |
| Spne_91 | CTCGACACATAACTCA | -22 | 16 | 43,8 | 59,6 | 243-258 |
| Spne_92 | CCTCGACACATAACT | -21,1 | 15 | 46,7 | 59,5 | 245-259 |
| Spne_93 | TACAACCATAGGTTATG | -21,5 | 17 | 35,3 | 53,8 | 276-292 |
| Spne_94 | CTACAACCATAGGTTATG | -22,9 | 18 | 38,9 | 56,9 | 276-293 |
| Spne_95 | TCTACAACCATAGGTTAT | -22,8 | 18 | 33,3 | 56,8 | 277-294 |
| Spne_96 | GTCTACAACCATAGGT | -21,9 | 16 | 43,8 | 57,8 | 280-295 |
| Spne_97 | AACAGCCCCT | -16,6 | 10 | 60 | 56,5 | 257-266 |
